# Competency Framework for Podiatric Medicine Training: A Validation Report Based on an Adapted E-Delphi Across Canada

**DOI:** 10.1177/23821205241234974

**Published:** 2024-02-26

**Authors:** Yassin Andoulsi, Olivier Hue, Martine Brousseau, James Hill, Joel Alleyne, Virginie Blanchette

**Affiliations:** 1 Department of Human Kinetics and Podiatric Medicine, 14847 Université du Québec à Trois-Rivières, Trois-Rivières, Canada; 2 Occupational Therapy Department, 14847 Université du Québec à Trois-Rivières, Trois-Rivieres, Canada; 3 Foot Care Institute, Windsor, Canada; 4 Canadian Podiatric Medical Association,Ontario, Canada; 5 Faculty of Information, University of Toronto, Toronto, Canada; 6 VITAM - Sustainable Health Research Centre, Québec, Canada

**Keywords:** podiatry, competency-based education, professional competence skills, Delphi technique, validation study

## Abstract

**OBJECTIVES:**

Competency-based medical education has been introduced into many health professional curricula. Based on CanMEDs, a framework has recently been developed for podiatric medicine education in Canada. This study aimed to validate the framework through a consensus of various podiatric medicine experts across Canada.

**METHODS:**

An adapted Delphi method was used for content validation. Two structured online questionnaires were used to gather expert opinions and agreement on the roles and core competencies described in the framework previously developed. The validation consensus threshold was set at a minimum of 80% agreement. The summary of comments and suggestions was used to reformulate certain items after the research team reached a consensus.

**RESULTS:**

Out of the 51 experts invited from the Canadian Podiatric Medicine Association, 19 completed the first-round questionnaire (mean podiatric experience = 24.5 years; standard deviation 17.6). After the first round, “Podiatric Expert”, “Communicator” and “Scholar” roles have been modified. After these modifications, a consensus was obtained at the second round completed by 13 experts. Overall, 95% of the experts agreed that the competency framework was relevant even if some indicators would need to be adapted to suit the requirements of each province and territory.

**CONCLUSION:**

This validated framework supports the excellence and the quality of our podiatric educational program. It also promotes the adoption of a uniform education of podiatrists in Canada and worldwide.

## Background

Competency-based medical education was developed several years ago.^1^ Its relevance has been demonstrated in many studies.^2–4^ It is postulated that it increases the quality and safety of healthcare by developing an educational system centered on the learner, which would contribute to the development of a healthcare system centered on the patient.^1^ This model can also enhance the clinical performance of health care providers.^5^

This approach was recently explored for podiatric medicine education using a modified Delphi method at our university.^6^ This resulted in the development of competency-based podiatric medicine education using the CanMEDS, a competency framework based on the work initiated by The Royal College of Physicians and Surgeons of Canada,^7^ which included seven roles (ie, podiatric expert, collaborator, communicator, health advocate, leader and manager, professional and scholar), 24 core competencies and 288 observable-related indicators.^6^

Content validation by external experts is an important step before implementation following knowledge product developed by a Delphi technique,^8^ particularly for the dissemination of our work across Canada or worldwide to improve the quality of podiatric medical education.^6^ Various methods (eg, Delphi technique and nominal group technique) for the validation of frameworks in medical education research exist, but these are poorly standardized.^9^ Therefore, our objective was to seek out consensus among podiatric medicine stakeholders across Canada in order to obtain controlled feedback to validate and enhance our framework.

## Methods

### Design, reporting guidelines, ethical considerations

Our competency framework development has been described elsewhere.^6^ This content validation was based on an adapted online Delphi technique (e-Delphi)^10^ as this eliminated the constraints of geographic location, and facilitated the organization and analysis of the data collected.^9,11,12^ This study is reported using CREDES (Conducting and REporting DElphi Studies) criteria,^8^ which promotes consistency and design reproducibility. Participation was voluntary, ethics approval was waived by the Institutional Review Board (Comité éthique de recherche avec les êtres humains of Université du Québec à Trois-Rivières) and the project respected the Tri-Council Policy Statement of Canada (in accordance with the Declaration of Helsinki).^13^ Electronic informed consent was obtained from the experts at the beginning of the questionnaire and the entire process was anonymous.

### Participants and recruitment

As it has been shown in previous studies that large panels generate low response rates and that more than 30 participants rarely improve results, the convenience sample size was estimated at between 12 and 24 experts; this was also to reduce the influence of rater aberration in the content validation task.^14,15^ Inclusion criteria were: 1) ability to read, understand and write in English; 2) obtained a diploma or degree related to podiatry/chiropody in Canada; 3) practice exclusively in Canada. The Canadian Podiatric Medical Association (CPMA) (JA) supported the experts’ identification, and the recruitment within their network by targeted podiatrists involved in continued education and profession development in Canada. For this validation study, an expert in podiatric medicine was someone who met our inclusion criteria. Potential participants were solicited by email from October to December 2022, with a detailed description of the study, the published framework, and the online questionnaire.

### Data collection and e-Delphi rounds

**Figure 1.**
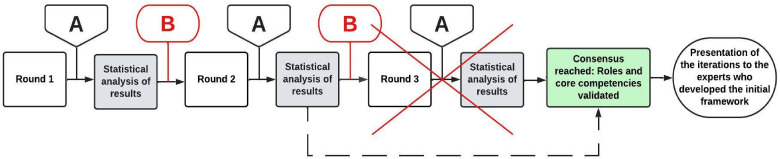
Summary of the adapted e-Delphi process. A: Two-week deadline for completion of the round; B: Team consensus related to relevancy, comprehensiveness, and clarity. Iteration of items; X: As consensus was reached at the end of Round 2, Round 3 was not necessary.

The team (VB, JH, OH) constructed a questionnaire using Qualtrics software (Qualtrics software, Provo, UT), knowledge user's input (JH, YA) and methodology expert (MB). This is not a validated questionnaire (Supplementary Appendix A). The questionnaire consisted of three sections: 1) participants’ characteristics; 2) level of agreement regarding the roles and core competencies using a 7-point Likert scale (1 [strongly disagree] to 7 [strongly agree]); and 3) general comments about the indicators or suggestions including the feasibility of implementation.^14^ Three rounds were expected for the e-Delphi process (Figure 1), detailed in Appendix B. The agreement threshold was set at 80% by the team as there is no standard.^14^ For each round, two reminders were sent to the participants to obtain the highest possible response rate and a two-week deadline was given to complete the questionnaire.

### Data analysis

Data analysis was performed using Qualtrics software (Qualtrics, Provo, UT) with descriptive statistics. A narrative synthesis was produced for the written content, which was evaluated by two researchers (VB, OH) and discussed with the entire team as necessary to move along the process.

## Results

The characteristics of the experts involved in this validation study are presented in Table 1. Most of the panel of experts were men practicing in the central region (ie, Québec or Ontario) who had a doctorate in podiatric medicine, which is the same degree as those who developed the initial framework. However, the majority had a surgical residency, which was not the case in the development phase.^6^ The mean experience in podiatric medicine of the panel was approximately 25 years.

**Table 1.** Participants’ characteristics.

Baseline characteristics per round^§^	Round 1	Round 2
*Responses Rate (total of 51 experts invited first round; n (%)*	19 (37)	13 (68)
*Main practice area, n (%)*		
West (AB, BC, MB, SK)	8 (42)	4 (24)
Centre (QC, ON)	9 (47)	7 (41)
East (PEI, NB, NC, NL)	2 (22)	2 (22)
North (NT, NU, YT)	0 (0)	0 (0)
*Gender, n (%)*		
Men	16 (84)	8 (62)
Women	3 (16)	3 (23)
Prefer not to say	0 (0)	0 (0)
Other	0 (0)	2 (15)
*Main podiatric accreditation, n (%)*		
DPM (USA/QC)	13 (68)	7(54)
D. Ch.	0 (0)	0 (0)
ASPM	0 (0)	0 (0)
BSc (Pod)	2 (11)	2 (15)
DE (podology)	0 (0)	0 (0)
BSc (Hons) Podiatry	3 (16)	3 (23)
Other^†^	1 (5)	1 (8)
*Highest level of education, n (%)*		
Medical degree including bachelor or equivalent	15 (79)	10 (77)
MSc or equivalent	3 (16)	2 (15)
PhD or equivalent	1 (5)	1 (8)
*Surgery residency completed, n (%)*	9 (47)	8 (62)
*Range of years of experience in podiatric medicine* ^‡^ *, mean (SD) [Min; Max]*	24.5 (17.6) [2; 49]	25.4 (13.7) [2; 49]

Notes. N,%: number, percentage; AB: Alberta; BC: British Columbia; MB: Manitoba, SK: Saskatchewan; QC: Québec; ON: Ontario; PEI: Prince Edward Island; NB: New Brunswick, NS: Nova Scotia; NL: Newfoundland and Labrador; NT: Northwest Territories; NU: Nunavut; YT: Yukon; DPM: Doctorate in podiatric Medicine; D.Ch: Doctorate in chiropodist; ASPM: Advance Standing in Podiatric Medicine; BSc (Pod): Bachelor of podiatric medicine; DE: Diplôme d'État en podologie; BSc (Hons) Podiatry: Fourth-year Bachelor of podiatric medicine; MSc: Master in Science; PhD: Philosophiæ doctor; SD : Standard Deviation; [Min; Max] : [Minimum; Maximum].

§ Round 3 was unnecessary.

† Not Specified.

‡ Ninety percent of experts are in full-time practice for both rounds; ^¶^This number is calculated from 37 participants included in the first round.

The response rate was 37% (19/51 invited participants) for the first round and 68% (13/19) for the second round (Table 1). As consensus was reached at the end of Round 2, the third round was unnecessary. As a result of this content validation (Table 2), the description of “Podiatric Expert” was slightly modified, along with a competency for this role. No changes were made to the “Collaborator”, “Health Advocate”, “Leader and Manager”, and “Professional” roles. Two competencies have been modified for the “Communicator” role, and a new indicator has been added to a competency under the “Scholar” role. The experts agreed (95%) that this framework could be relevant to continuing medical education even if some indicators may need adaptation across the provinces and territories.

**Table 2.** Adapted e-Delphi results.

Role^†^	Description / Core Competencies^†^	Round 1 Mean (SD)^‡^	Round 2 Mean (SD)	Iterations (in bold) post-validation supported by narrative comments
Podiatric Expert	Description of the role	5.47 (1.90)	6.28 (2.13)	Podiatrists are empowered under province-specific laws legislating the profession to perform “any procedure” to treat local foot conditions that are not systemic diseases. As podiatric experts, they are aware of the limits of their knowledge and skills and determine the pathology affecting the health of the patient's feet. They plan and apply the appropriate diagnostic and therapeutic examinations and provide necessary treatments according to recognized and proven standards of practice in accordance with current **evidence** related to podiatric medicine. The podiatrist knows how and when to refer. The environment in which podiatrists perform their professional services must be safe. The role of the podiatric expert is essential to the podiatrist's function and represents the central role overseeing the other six roles of the podiatric medicine competency framework, namely the communicator, collaborator, leader, health advocate, scholar and professional.
	Assess the patient's symptoms and general history.	6.63 (1.35)		
	Conduct a clinical assessment focused on the patient's needs.	6.68 (1.34)		
	Determine the pathology affecting the patient's foot condition.	6.53 (1.39)		
	Identify, plan, and carry out diagnostic and therapeutic interventions appropriate to the patient's needs.	5.53 (1.39)	6.12 (1.80)	Identify, plan, and carry out diagnostic and therapeutic interventions, **including referral pathways**, appropriate to the patient's needs.
Collaborator	Description of the role	6.21 (1.82)		
	Work efficiently with other podiatrists or health care professionals to foster collaboration and mutual understanding of the patient's needs.	6.58 (1.35)		
	Work with other health professionals to promote mutual understanding, manage differences and resolve conflicts.	6.26 (1.41)		
	Transition and transfer patient care to another podiatrist or health care professional in a safe manner to ensure continuity of care.	6.63 (1.35)		
	Ensure satisfaction with the collaborative work between the patient and the podiatrist.	6.26 (1.41)		
Communicator	Description of the role	5.84 (1.95)		
	Establish a trusting professional relationship with the patient, family and caregivers.	6.47 (1.35)		
	Gather and synthesize information relevant to the medical history by documenting the information and maintaining a record for each patient to ensure clinical decision-making.	6.58 (1.35)		
	Inform the patient, family and caregivers about the podiatric care provided.	5.21 (1.44)	6.28 (1.48)	Inform the patient (and the family and caregivers **with the patient's permission or appropriate authority**), about the podiatric care provided.
	Communicate in writing	5.54 (1.46)	6.00 (1.50)	Communicate in writing **integrating the implicit permission of the patient or appropriate authority**.
Health Advocate	Description of the role	5.89 (1.83)		
	Promote foot health and engage in the prevention of local foot conditions.	6.47 (1.35)		
	Promote access to podiatric care and advocate for improved care.	6.32 (1.42)		
Leader and Manager	Description of the role	6.05 (1.79)		
	Promote quality and innovation in the delivery of podiatric care.	6.47 (1.35)		
	Contribute to the proper functioning of the system.	6.16 (1.35)		
	Manage the development and planning of one's career, human and financial resources in the exercise of one's professional activities.	6.05 (1.39)		
Professional	Description of the role	5.95 (1.96)		
	Demonstrate a commitment to the patient through the application of best practices and adherence to ethical and deontological standards.	6.58 (1.35)		
	Demonstrate a commitment to society by recognizing and meeting its expectations for podiatric care.	6.16 (1.69)		
	Demonstrate a commitment to the profession through adherence to the standards, laws and regulations governing the practice of podiatric medicine and participation in the self-regulation of the profession.	6.58 (1.35)		
	Demonstrate a commitment to the health and well-being of podiatrists to support the delivery of optimal podiatric care to patients.	6.42 (1.39)		
Scholar	Description of the role	5.89 (1.65)		
	Engage in continuous improvement of professional activities through a process of continuing education.	6.42 (1.35)		
	Teach peers and other health professionals and the public.	6.05 (1.43)		
	Research, evaluate, and apply evidence in their field using a scientific approach.	6.16 (1.42)	5.96 (1.59)^±^	New indicator: **Determine the risk/benefit ratio of new data for patient health using the best available knowledge when evidence is very limited.**
	Contribute to the dissemination and creation of podiatric knowledge and practices applicable to podiatric medicine.	6.11 (1.33)		

Legend and abbreviations:

† The original framework with description of roles, competencies and indicators is available in Blanchette et al 2022.^6^

**^‡^** Mean on a maximum of 7. All items that did not meet the minimum threshold of 80% (5.6/7) went through to the second round.

± Agreement on the new indicator added after the first round.

## Discussion

This short report adds to the growing literature about education related to podiatric medicine.^6,16–18^ This validation has contributed to modifying the initial competency framework, taking into account expertise across Canada.^6^ This framework was presented to the experts who developed the initial framework, and this version will be implemented. The iterations supported medical reasoning to the “Podiatric Expert” and the “Scholar”, care trajectory to the “Podiatric Expert” and ethical consideration to the “Communicator” roles which are all in alignment with care quality and the Quintuple Aims.^19^

The expert panel was balanced geographically. However, as there were few podiatrists in the northern regions, we were unable to represent these areas. The experts solicited had more experience (25 years vs 18 years) and were more surgically trained (47% vs 37%) than at our institution.^6^ This is consistent with the fact that podiatrists outside Québec are often trained in English-speaking countries, mostly in the United States requiring residency, and Québec does not offer a residency program.^20^ Some provinces, particularly in the Western regions, require a surgical residency to practice or to perform certain procedures.^21^ There is also a gender disparity in the expert panel (16% women vs 37% men), but this is aligned with what it is observed in this profession in North America.^22^

This validation may support a common vision and language regarding the education of podiatrists in Canada, notwithstanding the differences in legislation and professional titles across Canada.^21^ This may encourage change and professional development to promote excellence and quality especially when no program accreditation is available.^23,24^ The experts agreed that this framework may support continuing education programs. This is seen in other professions.^25,26^ The CanMEDS framework can support potential learning needs associated with specific competencies and/or indicators. Perceived learning needs and challenges encountered in practice can provide insight into the selection and design of continuing education activities and empower the learner.^25^

Limitations to this study must be emphasized, as the type of sampling and the population does not necessarily represent all Canadian podiatrists, notably due to the wide variety of degrees and the fact that the experts were chosen by a specific network (CPMA). Our questionnaire was not validated. The first questionnaire was long (Supplementary Appendix A), which may have led to fatigue and less accurate responses. However, the results add value to our previous work, and the validated version represents a broader perspective to enhance the quality of our program and hopefully, the quality of podiatric care. This study may also serve as preliminary work for another similar study, but on an international scale.

## Conclusion

This validation process led to the enhancement of a framework that now takes into account the expertise of many professionals across Canada focused on foot care. This framework promotes the excellence and quality of our podiatry training program and, ultimately, foot care and prevention. By implementing it locally in Canada and, hopefully, internationally, it could foster a common vision and language for consistent training among podiatrists.

## Supplemental Material

sj-docx-1-mde-10.1177_23821205241234974 - Supplemental material for Competency Framework for Podiatric Medicine Training: A Validation Report Based on an Adapted E-Delphi Across Canada Supplemental material, sj-docx-1-mde-10.1177_23821205241234974 for Competency Framework for Podiatric Medicine Training: A Validation Report Based on an Adapted E-Delphi Across Canada by Yassin Andoulsi, Olivier Hue, Martine Brousseau, James Hill, Joel Alleyne and Virginie Blanchette in Journal of Medical Education and Curricular Development

sj-docx-2-mde-10.1177_23821205241234974 - Supplemental material for Competency Framework for Podiatric Medicine Training: A Validation Report Based on an Adapted E-Delphi Across Canada Supplemental material, sj-docx-2-mde-10.1177_23821205241234974 for Competency Framework for Podiatric Medicine Training: A Validation Report Based on an Adapted E-Delphi Across Canada by Yassin Andoulsi, Olivier Hue, Martine Brousseau, James Hill, Joel Alleyne and Virginie Blanchette in Journal of Medical Education and Curricular Development
