# Supplementary material for: Competency Framework for Podiatric Medicine Training: A Validation Report Based on an Adapted E-Delphi Across Canada
Source: J Med Educ Curric Dev. 2024 Feb 26;11:23821205241234974. doi: 10.1177/23821205241234974 (PMC10898292; doi:10.1177/23821205241234974)
Supplement: sj-docx-1-mde-10.1177_23821205241234974 - Supplemental material for Competency Framework for Podiatric Medicine Training: A Validation Report Based on an Adapted E-Delphi Across Canada [file sj-docx-1-mde-10.1177_23821205241234974.docx]

Appendix A

UQTR-CPMA podiatric medicine framework - Round 1

Début de bloc: Intro

Greetings,

You have been identified and selected by the CPMA as an expert in the field of podiatric medicine to provide your input for the development of a competency framework which is based on the CanMEDS Roles (i.e. expert, collaborator, communicator, health advocate, leader, professional, scholar)*.

You are invited to participate, on a voluntary basis, to answer this first questionnaire that contains 42 questions. Please note that it is possible to pause the survey and complete it later from where it was left only if the same browser is used. This questionnaire may be long, but the other one will be shorter.

The first questionnaire has 3 sections. The first section is related to the baseline characteristics of the respondent (i.e. you as an expert). The second section is related to core competencies where you are asked to express your agreement on a scale of 1 (strongly disagree) to 7 (strongly agree).
The third section consists of 4 open-ended questions asking if you 1) have any comments or suggestions, 2) have any additional information to add (eg. additional competencies), and 3) think that such a competency framework could be applicable in your daily podiatry practice and 4) relevant or adapted to continuing medical education (CME) in podiatry or chiropody. 

The entire process is confidential, and you will never be specifically identified on your answers. You will provide your consent by answering the questionnaire. The data will be submitted at the Journal of Medical Education and Curricular Development at the first place and then to another journal as required. If you do not agree to participate, please do not answer the questionnaire.

*To learn more about CanMEDS Roles, additional information will be provided at the end of this questionnaire.

Q1 Consent

- Yes (1)
- No (2)

Fin de bloc: Intro

Début de bloc: Part 1 - Demographic data

Q2. Identify your province or territory of main practice/job

- Alberta (1)
- British Columbia (2)
- Prince Edward Island (3)
- Manitoba (4)
- New Brunswick (5)
- Nova Scotia (6)
- Ontario (7)
- Quebec (8)
- Saskatchewan (9)
- Newfoundland and Labrador (10)
- Northwest Territories (11)
- Nunavut (12)
- Yukon (13)

Q3. To which gender do you identify ?

- Male (1)
- Female (2)
- Non-binary / third gender (3)
- Prefer not to say (4)
- Other : (5) __________________________________________________

Q4. What is your main podiatric accreditation?

- Doctor of Podiatric Medicine (DPM) - USA or QC (1)
- Diploma in Chiropody (D.Ch.) (2)
- American Society of Podiatric Medicine (ASPM) (3)
- Bachelor of podiatric medicine BSc (Pod) (4)
- Diplôme d'Etat de pédicure-podologue (DE) (5)
- BSc (Hons) Podiatry (6)
- Other : (7) __________________________________________________

Q5. What is your highest level of education?

- Medical degree (MD, D.Ch., DPM…) including bachelor or equivalent (1) __________________________________________________
- Master (MSc) or equivalent (2)
- Doctor in philosophy (PhD) or equivalent (3)

Q6. Have you completed a surgical residency?

- Yes (1)
- No (2)

Q7. How long have you been in practice?
Please enter the corresponding number of years of practice.

________________________________________________________________

Q8. Which of the following represents the majority of your practice ?
 Please use the sliders to indicate the percentage of time allocated to your practice.

|  | 0 | 10 | 20 | 30 | 40 | 50 | 60 | 70 | 80 | 90 | 100 |
| --- | --- | --- | --- | --- | --- | --- | --- | --- | --- | --- | --- |

| Full-time clinical practice (majority of the practice) () | 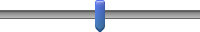 |
| --- | --- |
| Part-time clinical practice (practice in another field simultaneously) () | 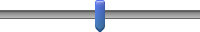 |
| Full-time research () | 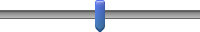 |
| Part-time research () | 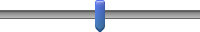 |
| Full-time teaching () | 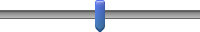 |
| Part-time teaching () | 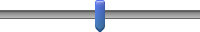 |

Fin de bloc: Part 1 - Demographic data

Début de bloc: Part 2 - Core competencies

**Intro - Part 2**
A core competency is defined as the functional adequacy and capacity to integrate knowledge and skills with attitudes and values into the specific context of practice.

In this section of the questionnaire, you will be presented with 7 roles and corresponding core competencies that we believe a podiatrist should acquire upon graduation and apply in practice.
 
For every role description and core competency you will have your level of agreement on a scale of 1 (strongly disagree) to 7 (strongly agree). You will also have the opportunity to provide comments and/or suggestions after each role.

Q9. *Podiatric Expert*

 On a scale of 1 (strongly disagree) to 7 (strongly agree), how would you rate your level of agreement regarding the role of the podiatric expert described below?

 *Podiatrist are empowered under province-specific laws legislating the profession to perform "any procedure" to treat local foot conditions that are not systemic diseases. As podiatric experts, they are aware of the limits of their knowledge and skills and determine the pathology affecting the health of the patient's feet. They plan and apply the appropriate diagnostic and therapeutic examinations and provide the necessary treatments according to recognized and proven standards of practice in accordance with current data related to podiatric medicine. The environment in which podiatrists perform their professional services must be safe. The role of the podiatric expert is essential to the podiatrist’s function and represents the central role overseeing the other six roles of the podiatric medicine competency framework namely the communicator, collaborator, leader, health advocate, scholar and professional.*

- (1) Strongly disagree (1)
- (2) Disagree (2)
- (3) Somewhat disagree (3)
- (4) Neither agree or disagree (4)
- (5) Somewhat agree (5)
- (6) Agree (6)
- (7) Strongly agree (7)

Q10. Do you have any comments regarding the description of the role of the podiatric expert?

________________________________________________________________

Q11. The role of the podiatric expert is characterized by 4 core competencies. Please indicate your level of agreement with each core competency described below.

  As podiatric experts, we should be able to:

|  | **1  Strongly disagree** (1) | **2  Disagree** (2) | **3  Somewhat disagree** (3) | **4  Neither agree or disagree** (4) | **5  Somewhat agree** (5) | **6  Agree** (6) | **7 Strongly agree** (7) |
| --- | --- | --- | --- | --- | --- | --- | --- |
| 1. Assess the patient's symptoms and general history. (1) |  |  |  |  |  |  |  |
| 2. Conduct a clinical assessment focused on the patient's needs. (2) |  |  |  |  |  |  |  |
| 3. Determine the pathology affecting the patient's foot condition. (3) |  |  |  |  |  |  |  |
| 4. Identify, plan, and carry out diagnostic and therapeutic interventions appropriate to the patient's needs. (4) |  |  |  |  |  |  |  |

Q12. Do you have any comments regarding the core competencies characterizing the role of the podiatric expert?

________________________________________________________________

Q13. *Collaborator*

 On a scale of 1 (strongly disagree) to 7 (strongly agree), how would you rate your level of agreement regarding the role of the collaborator described below?

 *Podiatrists are primary health professionals. They must collaborate effectively with other health professionals in an effective way to provide appropriated quality care satisfying the patient’s needs. This may be accomplished by referring the patient to another health care professional or ensuring direct and continuous communication with another professional to ensure appropriate continuity of health care. Podiatrists must be aware of the limitation to their level of competence or the efficacy of their services. If the patient’s interest and needs require it, podiatrists must refer the patient to another health professional with the patient’s authorization. Collaborative decision-making among service providers requires above all a full understanding of their individual roles, the optimization of patient care and the allocation of responsibilities. In addition to collaborating with their peers, podiatrists must also collaborate with their patient by including the patient’s perspective into the decision-making process of the treatment plan. Podiatrists must also respect a patient’s choice to consult another podiatrist or another health professional. Podiatrist must also respect the patient’s choice in having their care executed by another professional.*

- (1) Strongly disagree (1)
- (2) Disagree (2)
- (3) Somewhat disagree (3)
- (4) Neither agree or disagree (4)
- (5) Somewhat agree (5)
- (6) Agree (6)
- (7) Strongly agree (7)

Q14. Do you have any comments regarding the description of the role of the collaborator?

Q15. The role of the collaborator is characterized by 4 core competencies. Please indicate your level of agreement with each core competency described below.

As collaborators, we should be able to:

|  | **1  Strongly disagree** (1) | **2  Disagree** (2) | **3  Somewhat disagree** (3) | **4  Neither agree or disagree** (4) | **5  Somewhat agree** (5) | **6  Agree** (6) | **7  Strongly agree** (7) |
| --- | --- | --- | --- | --- | --- | --- | --- |
| 1. Work efficiently with other podiatrists or health care professionals to foster collaboration and mutual understanding of the patient's needs. (5) |  |  |  |  |  |  |  |
| 2. Work with other health professionals to promote mutual understanding, manage differences and resolve conflicts. (6) |  |  |  |  |  |  |  |
| 3. Transition and transfer patient care to another podiatrist or health care professional in a safe manner to ensure continuity of care. (7) |  |  |  |  |  |  |  |
| 4. Ensure satisfaction with the collaborative work between the patient and the podiatrist. (8) |  |  |  |  |  |  |  |

Q16. Do you have any comments regarding the core competencies characterizing the role of the collaborator?

________________________________________________________________

|  |
| --- |

Q17. *Communicator*

 On a scale of 1 (strongly disagree) to 7 (strongly agree), how would you rate your level of agreement regarding the role of the communicator described below?

 *Podiatrists must seek to establish and maintain a professional relationship of mutual trust with their patients and their relatives since a disorder affects not only the patient, but also their relatives (e.g., family, friends, caregivers). Podiatrists must be both professional and empathetical in their physical, mental, and emotional interaction with both patients and relatives. Podiatrists seek full knowledge of the state of health of their patient and their patient’s needs even if their field of activities is directed to treating local foot conditions. Podiatrists must communicate effectively and in a structured way, so that the patient understands the nature of problems identified, the risks and the benefits associated with each of the proposed treatments, and thus can give explicit and informed consent. The treatment plan must be explained in a way that considers the patient’s medical history, lifestyle, needs and socioeconomic situation. Podiatrists must inform their patient of the limit of their expertise, in cases where the patient must be referred to another medical specialty.*

- (1) Strongly disagree (1)
- (2) Disagree (2)
- (3) Somewhat disagree (3)
- (4) Neither agree or disagree (4)
- (5) Somewhat agree (5)
- (6) Agree (6)
- (7) Strongly agree (7)

Q18. Do you have any comments regarding the description of the role of the communicator?

________________________________________________________________

Q19. The role of the communicator is characterized by 4 core competencies. Please indicate your level of agreement with each core competency described below.

 As communicators, we should be able to:

|  | **1  Strongly disagree** (1) | **2  Disagree** (2) | **3  Somewhat disagree** (3) | **4  Neither agree or disagree** (4) | **5  Somewhat agree** (5) | **6  Agree** (6) | **7  Strongly agree** (7) |
| --- | --- | --- | --- | --- | --- | --- | --- |
| 1. Establish a trusting professional relationship with the patient, family and caregivers. (1) |  |  |  |  |  |  |  |
| 2. Gather and synthesize information relevant to the medical history by documenting the information and maintaining a record for each patient to ensure clinical decision-making. (2) |  |  |  |  |  |  |  |
| 3. Inform the patient, family and caregivers about the podiatric care provided. (3) |  |  |  |  |  |  |  |
| 4. Communicate in writing. (4) |  |  |  |  |  |  |  |

Q20. Do you have any comments regarding the core competencies characterizing the role of the communicator?

________________________________________________________________

Q21. *Health Advocate*
 On a scale of 1 (strongly disagree) to 7 (strongly agree), how would you rate your level of agreement regarding the role of the health advocate described below?

 *Podiatrists contribute to the development of the provision of foot care to the general public and in clinical situations. Podiatrists know how to communicate, educate and share their knowledge and expertise with patients, their family and their caregivers. Podiatrists promote their profession by informing and educating other health professionals to promote effective interprofessional collaboration and also ensure patient follow-up. Podiatrists promote education and information about podiatric medicine.*

- (1) Strongly disagree (1)
- (2) Disagree (2)
- (3) Somewhat disagree (3)
- (4) Neither agree or disagree (4)
- (5) Somewhat agree (5)
- (6) Agree (6)
- (7) Strongly agree (7)

Q22. Do you have any comments regarding the description of the role of the health advocate?

________________________________________________________________

Q23. The role of the health advocate is characterized by 2 core competencies. Please indicate your level of agreement with each core competency described below.

 As health advocates, we should be able to:

|  | **1  Strongly disagree** (1) | **2  Disagree** (2) | **3  Somewhat disagree** (3) | **4  Neither agree or disagree** (4) | **5  Somewhat agree** (5) | **6  Agree** (6) | **7  Strongly agree** (7) |
| --- | --- | --- | --- | --- | --- | --- | --- |
| 1. Promote foot health and engage in the prevention of local foot conditions. (1) |  |  |  |  |  |  |  |
| 2. Promote access to podiatric care and advocate for improved care. (2) |  |  |  |  |  |  |  |

Q24 Q24. Do you have any comments regarding the core competencies characterizing the role of the health advocate?

________________________________________________________________

Q25. *Leader and Manager*

 On a scale of 1 (strongly disagree) to 7 (strongly agree), how would you rate your level of agreement regarding the role of the leader and manager described below?

 *Podiatrists ensure that the podiatric medical acts they perform comply with scientific and professional standards. Podiatrists ensure that their clinic or their place of practice is equipped in accordance with these standards and that the persons under their supervision know these standards and respect them at all times. They contribute to the evolution, development and practice of their profession by taking care to inform the other health professionals about their field of expertise. Podiatrists participate in making decisions in collaboration with other health professionals to contribute to the evolution of the provision of health care. Podiatrists also work to maintain a healthy work environment that is respectful to their personnel and their patients. Podiatrists take responsibility for time management, career management and professional practice management.*

- (1) Strongly disagree (1)
- (2) Disagree (2)
- (3) Somewhat disagree (3)
- (4) Neither agree or disagree (4)
- (5) Somewhat agree (5)
- (6) Agree (6)
- (7) Strongly agree (7)

Q26. Do you have any comments regarding the description of the role of the leader and manager?

________________________________________________________________

Q27. The role of the leader and manager is characterized by 3 core competencies. Please indicate your level of agreement with each core competency described below.

  As leaders and managers, we should be able to:

|  | **1  Strongly disagree** (1) | **2  Disagree** (2) | **3  Somewhat disagree** (3) | **4  Neither agree or disagree** (4) | **5  Somewhat agree** (5) | **6  Agree** (6) | **7  Strongly agree** (7) |
| --- | --- | --- | --- | --- | --- | --- | --- |
| 1. Promote quality and innovation in the delivery of podiatric care. (1) |  |  |  |  |  |  |  |
| 2. Contribute to the proper functioning of the system. (2) |  |  |  |  |  |  |  |
| 3. Manage the development and planning of one's career, human and financial resources in the exercise of one's professional activities. (3) |  |  |  |  |  |  |  |

Q28. Do you have any comments regarding the core competencies characterizing the role of the leader and manager?

________________________________________________________________

Q29. *Professional*

 On a scale of 1 (strongly disagree) to 7 (strongly agree), how would you rate your level of agreement regarding the role of the professional described below?

 *As professionals, podiatrists have the duty to promote foot health, among both individuals and the collectivity. Podiatrists must subject their practice to the standards of their profession and the Code of ethics of podiatrists and respect the province-specific laws legislating the profession. As health professionals, podiatrists must apply every effort to meet society’s expectations from the profession. They undertake to offer clinical competency, maintain that competency and practice in accordance with ethical standards. They also commit to demonstrate values such as integrity, honesty, altruism, respect for patients without judgment or discrimination, and to act transparently with respect to potential conflicts of interest.*

- (1) Strongly disagree (1)
- (2) Disagree (2)
- (3) Somewhat disagree (3)
- (4) Neither agree or disagree (4)
- (5) Somewhat agree (5)
- (6) Agree (6)
- (7) Strongly agree (7)

Q30. Do you have any comments regarding the description of the role of the professional?

________________________________________________________________

Q31. The role of the professional is characterized by 4 core competencies. Please indicate your level of agreement with each core competency described below.

As professionals, we should be able to:

|  | **1  Strongly disagree** (1) | **2  Disagree** (2) | **3  Somewhat disagree** (3) | **4  Neither agree or disagree** (4) | **5  Somewhat agree** (5) | **6  Agree** (6) | **7  Strongly agree** (7) |
| --- | --- | --- | --- | --- | --- | --- | --- |
| 1. Demonstrate a commitment to the patient through the application of best practices and deontological ethics. (1) |  |  |  |  |  |  |  |
| 2. Demonstrate a commitment to society by recognizing and meeting its expectations for podiatric care. (2) |  |  |  |  |  |  |  |
| 3. Demonstrate a commitment to the profession through adherence to the standards, laws and regulations governing the practice of podiatric medicine and participation in the self-regulation of the profession. (3) |  |  |  |  |  |  |  |
| 4. Demonstrate a commitment to the health and well-being of podiatrists to support the delivery of optimal podiatric care to patients. (6) |  |  |  |  |  |  |  |

Q32. Do you have any comments regarding the core competencies characterizing the role of professional?

________________________________________________________________

Q33. *Scholar*

 On a scale of 1 (strongly disagree) to 7 (strongly agree), how would you rate your level of agreement regarding the role of the scholar described below?

 *As scholars, podiatrists demonstrate constant commitment to the excellence of podiatric medicine so that they can provide quality care. They pursue this excellence through continuing education, research and teaching, all in accordance with a rigorous scientific approach. Podiatrists aim to improve their knowledge and competencies through available training. They share knowledge, compare methods and the results obtained with peers, and seek feedback to maintain quality of care and preserve patient safety. They collaborate in research and share knowledge, expertise and experience with the members of their profession and students. Podiatrists show their mastery of the practice of their profession by using an approved scientific approach, based on evidence. They evaluate and apply evidence as part of their practice (evidence-based practice). They can recognize the limits, uncertainties and gaps in their profession and formulate research questions that seek to improve it.*

- (1) Strongly disagree (1)
- (2) Disagree (2)
- (3) Somewhat disagree (3)
- (4) Neither agree or disagree (4)
- (5) Somewhat agree (5)
- (6) Agree (6)
- (7) Strongly agree (7)

Q34. Do you have any comments regarding the description of the role of the scholar?

________________________________________________________________

Q35. The role of the scholar is characterized by 4 core competencies. Please indicate your level of agreement with each core competency described below.

 As scholars, we should be able to:

|  | **1  Strongly disagree** (1) | **2  Disagree** (2) | **3  Somewhat disagree** (3) | **4  Neither agree or disagree** (4) | **5  Somewhat agree** (5) | **6  Agree** (6) | **7  Strongly agree** (7) |
| --- | --- | --- | --- | --- | --- | --- | --- |
| 1. Engage in continuous improvement of professional activities through a process of continuing education. (1) |  |  |  |  |  |  |  |
| 2. Teach peers and other health professionals and the public. (2) |  |  |  |  |  |  |  |
| 3. Research, evaluate, and apply evidence in his/her field using a scientific approach. (3) |  |  |  |  |  |  |  |
| 4. Contribute to the dissemination and creation of podiatric knowledge and practices applicable to podiatric medicine. (6) |  |  |  |  |  |  |  |

Q36. Do you have any comments regarding the core competencies characterizing the role of scholar?

________________________________________________________________

Fin de bloc: Part 2 - Core competencies

Début de bloc: Part 3 - Open-ended questions

Q37. Do you have any comments and/or suggestions?

________________________________________________________________

Q38. Do you have anything else to add regarding the roles and/or the core competencies described throughout the questionnaire?

________________________________________________________________

Q39. Do you think that such a competency framework could be applicable to you as a podiatrist/chiropodist in practice?

- Yes (1)
- No (3)
- Maybe (2)

Q40. Comments regarding the previous question.

________________________________________________________________

Q41. Do you think that such a competency framework could be relevant or adapted to continuing medical education (CME) in podiatry/chiropody?

- Yes (1)
- No (3)
- Maybe (2)

Q42. Comments regarding the previous question.

________________________________________________________________

Fin de bloc: Part 3 - Open-ended questions
