# Supplementary material for: Competency Framework for Podiatric Medicine Training: A Validation Report Based on an Adapted E-Delphi Across Canada
Source: J Med Educ Curric Dev. 2024 Feb 26;11:23821205241234974. doi: 10.1177/23821205241234974 (PMC10898292; doi:10.1177/23821205241234974)
Supplement: sj-docx-2-mde-10.1177_23821205241234974 - Supplemental material for Competency Framework for Podiatric Medicine Training: A Validation Report Based on an Adapted E-Delphi Across Canada [file sj-docx-2-mde-10.1177_23821205241234974.docx]

**Appendix B**

1.1 First e-Delphi Round

The duration of the first questionnaire was estimated at 50 minutes. Experts received an invitation to participate by e-mail from CPMA staff for each round. Data were analyzed after data collection by the research team (YA, VB and OH). Comments and suggestions from the experts were summarized. Items for which there was no consensus (<80%) were reworded or certain information based on comments and suggestions was added by the research team, without altering the meaning, considering that the item had been approved within the initial framework. The aim was to clarify or add new content that might have been omitted in the initial framework. Adapted items and new items suggested were brought back to the second round of e-Delphi.

1.2. Second E-Delphi Round

The duration of the second questionnaire was estimated at five minutes. Experts who participated in the first round of the e-Delphi were invited to complete the questionnaire. A report of the results of the first round was also sent altogether with the questionnaire to provide feedback to the experts (the anonymity of this information was preserved to avoid influences on the honesty of the answers provided). Data were analyzed after collection and the same method was used as for the first round. Adapted items were brought back to the third round of e-Delphi but was not required in our study.

1.3. Third e-Delphi Round

In this third and final round, experts who responded to the second round were invited. This questionnaire would have only retained the items for which consensus was not reached (with clarifications and feedback provided) at the second round. The item below the agreement threshold would have been rejected and would have been considered as non-validated.

1.4 Return to the Initial Experts

The research team plans to discuss the non-validated items with the experts who originally developed the framework to find solutions in response to the results. In any case, the results were presented to the experts to ensure the acceptance of the minor modifications and the integration of the item that has been added.
